# Editorial: Case reports in intensive care medicine 2025

**DOI:** 10.3389/fmed.2025.1747490

**Published:** 2025-12-04

**Authors:** Cheng Zhu, Bin Lin, Zhongheng Zhang, Yuetian Yu

**Affiliations:** 1 Department of Disease Prevention and Control, Ruijin Hospital, Shanghai Jiao Tong University, School of Medicine, Shanghai, China; 2 Key Laboratory of Intelligent Pharmacy and Individualized Therapy of Huzhou, Huzhou, China; 3 Department of Pharmacy, Changxing People's Hospital, Changxing Branch, Second Affiliated Hospital of Zhejiang University School of Medicine, Huzhou, China; 4 Department of Emergency Medicine, Sir Run-Run Shaw Hospital, Zhejiang University School of Medicine, Hangzhou, China; 5 Provincial Key Laboratory of Precise Diagnosis and Treatment of Abdominal Infection, Sir Run Run Shaw Hospital, Zhejiang University School of Medicine, Hangzhou, China; 6 Department of Critical Care Medicine, Renji Hospital, School of Medicine, Shanghai Jiao Tong University, Shanghai, China

**Keywords:** intensive care medicine, case reports, personalized medicine, heterogeneity, clinical phenotypes, artificial intelligence

The landscape of Intensive Care Medicine (ICM) is one of constant tension between the urgent need for standardized, evidence-based protocols and the profound biological and clinical heterogeneity of the patients we treat. The presentations of critically ill patients are a complex tapestry woven from unique genetic backgrounds, diverse comorbidities, varying sites and types of infection, and vastly different host immune responses. This inherent variability is the fundamental challenge that confronts every intensivist at the bedside (1). While large-scale randomized controlled trials (RCTs) and meta-analyses provide the essential scaffolding for our practice, they often obscure the individual patient narrative, generating population-level evidence that may not fit the unique circumstances of a specific case. It is within this gap between the generalizable knowledge of RCTs and the nuanced reality of individual patient care that the humble case report finds its powerful and enduring voice.

This Frontiers in Medicine Research Topic, “*Case reports in intensive care medicine 2025*,” was conceived with this very principle in mind. Our goal was to curate a collection of clinical narratives that not only inform and educate but also illuminate the path toward a more personalized, precise future for critical care. The 11 manuscripts accepted for this Research Topic serve as compelling testaments to the diversity of critical illness. They chronicle diagnostic dilemmas, innovative therapeutic maneuvers, unexpected complications, and hard-won clinical victories. More importantly, they argue persuasively for an evolution in our approach from a model of “one-size-fits-all” to one of “right treatment, for the right patient, at the right time.”

## The unyielding challenge of heterogeneity in the ICU

The quest for standardization in ICM is understandable. Protocols for sepsis management, ventilator liberation, and nutrition delivery bring order to chaos and have undoubtedly improved overall outcomes. However, the limitations of this approach become starkly apparent when faced with the biological reality of critical illness (2). Two patients with identical Sequential Organ Failure Assessment (SOFA) scores may have arrived at that point through entirely different pathophysiological pathways and may possess dramatically different capacities for recovery (3).

This heterogeneity is most vividly exemplified in sepsis, a core focus of modern ICM. Once considered a monolithic entity, sepsis is now recognized as a syndrome encompassing multiple distinct endotypes and subphenotypes (4). Through unsupervised machine learning analyses of large clinical and transcriptomic datasets, researchers have consistently identified categories such as the “hyperinflammatory” subphenotype (characterized by high vasopressor demand, metabolic acidosis, and inflammatory markers) and the “immunosuppressive” subphenotype (marked by lymphocyte exhaustion and a high risk of secondary infection). These subphenotypes demonstrate markedly different responses to therapy; for instance, the hyperinflammatory group may derive harm from liberal fluid resuscitation, while the immunosuppressive group might benefit from immunostimulatory agents (5).

The cases in this Research Topic reflect this heterogeneity beyond sepsis. We encounter patients with rare autoimmune etiologies of respiratory failure, unusual presentations of toxicological emergencies, and complex post-operative courses defying standard management algorithms. One case details the challenge of managing a patient with a rare genetic disorder who develops acute respiratory distress syndrome (ARDS), where conventional ventilator strategies prove insufficient or even detrimental. Another describes a cryptic source of infection in an immunocompromised host, where the usual microbiological suspects are absent, demanding a broader, more imaginative diagnostic approach (6). These narratives force us to acknowledge that our protocols are starting points, not destinations. They are maps that require constant interpretation and deviation based on the unique terrain of each patient.

## Case reports as the bedrock of experiential learning and incremental progress

In an era dominated by the hierarchy of evidence, case reports are sometimes unfairly relegated to a lower tier. This perspective overlooks their multifaceted and critical role in the advancement of ICM. They are not merely “anecdotes” but are, in fact, the fundamental units of clinical experience, serving several indispensable functions:

1. Catalysts for Discovery and Hypothesis Generation: Many groundbreaking advances in medicine began with a single, astute observation. A case report documenting an unexpected drug effect, a novel complication of a device, or a successful application of a therapy in a new context can generate hypotheses that fuel subsequent rigorous research. The report in this Research Topic on the use of a specific antidote for an uncommon poisoning, for instance, provides a template for management that may not be covered in any guideline but could be life-saving for a future patient.
2. Enhancing Diagnostic Acumen: Critically ill patients often present with constellations of symptoms that are diagnostically ambiguous. Case reports that detail rare mimics of common conditions, such as a non-infectious condition masquerading as septic shock, or an unusual metabolic disorder presenting as unexplained lactic acidosis, and they serve as vital educational tools. They broaden the differential diagnosis for clinicians worldwide, fostering a culture of intellectual curiosity and diagnostic rigor at the bedside.
3. Illustrating Therapeutic Innovation and Nuance: While RCTs tell us whether a treatment works on average, case reports often show us how it can be applied, adapted, and tailored in complex, real-world scenarios. A case in this topic detailing the meticulous, PK/PD-guided dosing of antibiotics in a patient with augmented renal clearance and septic shock provides a masterclass in personalized antimicrobial therapy. It moves beyond the trial protocol of fixed-dosing and demonstrates the art and science of optimizing drug exposure for an individual, which is a cornerstone of antimicrobial stewardship (7, 8).
4. Documenting Pitfalls and Fostering Safety: Not all cases end in success. Reporting on unexpected treatment failures, diagnostic errors, or rare adverse events is a courageous and essential form of collective learning. By analyzing what went wrong, we build systems and cognitive checks to prevent future occurrences. A case describing a complication related to a specific mode of extracorporeal membrane oxygenation (ECMO), for example, alerts other centers to a potential risk, enhancing safety for patients globally (9).

The 11 cases in this Research Topic collectively form a rich repository of this type of practical, frontline knowledge. They are a testament to the clinical wisdom that is accumulated not only through large datasets but also through the deep, reflective engagement with individual human stories.

## The compass for the future: navigating toward truly individualized care

The narratives presented here are more than just historical accounts, they are signposts pointing toward the future of ICM. The journey toward personalized critical care will be propelled by the integration of several key technological and philosophical shifts, many of which are prefigured in the cases of this Research Topic.

1. From Syndromes to Subphenotypes: The future lies in moving from classifying patients by broad syndromes to rapidly identifying their specific biological subphenotype. This will involve point-of-care technologies such as rapid transcriptomic profiling, metabolomic analysis, and focused genetic testing. Imagine a future where within hours of ICU admission, we can determine not just that a patient has sepsis, but that they have an immunosuppressive endotype with specific monocyte dysfunction, guiding us toward targeted immunostimulation rather than blanket, and potentially harmful, immunosuppression.
2. The Rise of the Digital Guardian: The vast, continuous streams of data generated in the ICU from vital signs and ventilator waveforms to laboratory results and nursing notes are beyond the integrative capacity of the human brain. Artificial intelligence (AI) and machine learning (ML) algorithms are poised to become indispensable partners. They can analyze this data in real-time to predict clinical deterioration earlier than current scoring systems, identify subtle subphenotypes, and even recommend personalized interventions. An AI system could, for example, analyze a patient's hemodynamic profile, inflammatory markers, and medication history to suggest the optimal vasopressor, its dose, and the ideal timing for antibiotic redosing based on real-time PK modeling (10).
3. Pharmacogenomics and Advanced Therapeutic Drug Monitoring (TDM): The paradigm of “one dose fits all” is particularly dangerous in the critically ill, whose pharmacokinetics are wildly altered by capillary leak, organ dysfunction, and extracorporeal circuits. The future entails routine TDM for a much wider array of drugs, particularly antibiotics, coupled with pharmacogenomic data (11, 12). Knowing a patient's genetic predisposition for metabolizing sedatives or responding to vasoactive agents will allow for truly bespoke pharmacotherapy from the outset.
4. Dynamic, Biomarker-Driven Treatment Courses: The rigid, calendar-based duration of antibiotic therapy or ventilator support is increasingly recognized as obsolete. The future is dynamic, guided by serial biomarkers. The use of procalcitonin to guide antibiotic discontinuation is an early example. Future strategies will incorporate a panel of biomarkers to answer the pivotal question daily: “Does this patient still need this specific intervention?” This aligns perfectly with the principles of antimicrobial de-escalation and ventilator liberation, ensuring therapies are applied only as long as they are beneficial (13, 14).

The cases in this Research Topic, in their detailed documentation of individual patient trajectories, underscore the necessity of this evolution. They show us clinicians thinking on their feet, adapting to new information, and crafting bespoke management plans. They are, in essence, early, human-powered examples of the personalized medicine we aim to systematize with technology.

## Conclusion

The case reports compiled in “*Case reports in intensive care medicine 2025*” are far more than isolated clinical curiosities. They are vital threads in the rich fabric of our specialty's knowledge. They remind us that the art of healing in the ICU lies in balancing the robust, population-derived evidence of our guidelines with the deep, empathetic, and nuanced understanding of the individual before us.

As Guest Editors, we extend our deepest gratitude to the authors who have generously shared their experiences, challenges, and insights. It is our sincere hope that this Research Topic will not only serve as a valuable educational resource but also inspire a renewed appreciation for the narrative in medicine. The path to precision critical care is a long and complex one, but each carefully observed and documented case report brings us a step closer. By listening to the stories of our patients, we learn not only how to be better doctors but also how to build a more intelligent, responsive, and ultimately more human, system of care for the most vulnerable among us.
